# Advancing healthcare through multimodal data fusion: a comprehensive review of techniques and applications

**DOI:** 10.7717/peerj-cs.2298

**Published:** 2024-10-30

**Authors:** Jing Ru Teoh, Jian Dong, Xiaowei Zuo, Khin Wee Lai, Khairunnisa Hasikin, Xiang Wu

**Affiliations:** 1 Department of Biomedical Engineering, University of Malaya, Kuala Lumpur, Malaysia; 2 China Electronics Standardization Institute, Beijing, China; 3 Department of Psychiatry, The Affiliated Xuzhou Oriental Hospital of Xuzhou Medical University, Xuzhou, Jiangsu, China; 4 Faculty of Engineering, Centre of Intelligent Systems for Emerging Technology (CISET), Kuala Lumpur, Malaysia; 5 Institute of Medical Information Security, Xuzhou, Jiangsu, China

**Keywords:** Multimodal data fusion, Healthcare, Medical images, EHR, Early fusion, Intermediate fusion, Late fusion, Patient care, Decision making, Challenges

## Abstract

With the increasing availability of diverse healthcare data sources, such as medical images and electronic health records, there is a growing need to effectively integrate and fuse this multimodal data for comprehensive analysis and decision-making. However, despite its potential, multimodal data fusion in healthcare remains limited. This review paper provides an overview of existing literature on multimodal data fusion in healthcare, covering 69 relevant works published between 2018 and 2024. It focuses on methodologies that integrate different data types to enhance medical analysis, including techniques for integrating medical images with structured and unstructured data, combining multiple image modalities, and other features. Additionally, the paper reviews various approaches to multimodal data fusion, such as early, intermediate, and late fusion methods, and examines the challenges and limitations associated with these techniques. The potential benefits and applications of multimodal data fusion in various diseases are highlighted, illustrating specific strategies employed in healthcare artificial intelligence (AI) model development. This research synthesizes existing information to facilitate progress in using multimodal data for improved medical diagnosis and treatment planning.

## Introduction

Automation in healthcare processes through the application of artificial intelligence (AI) has the capacity to bring transformative changes. However, in most AI applications, the predominance reliance on unimodal data such as computed tomography (CT) scans, magnetic resonance imaging (MRI), X-rays images *etc*. presents unique challenges in modern healthcare applications. These models frequently fail to incorporate crucial complementary data sources and various modalities, which limits their capacity to provide comprehensive insights (El-Sappagh et al., 2020; Moshawrab et al., 2023).

Healthcare AI applications are predominantly dominated by single-task models that rely on singular data types, lacking comprehensive clinical context. This contrasts with the holistic methods favored by clinicians and signifies a missed opportunity. Neglecting to utilize multimodal systems, which integrate multiple data modalities and interdependent tasks, hinders treatment efficacy and diagnostic accuracy (Acosta et al., 2022; El-Sappagh et al., 2020). Despite their potential for more accurate and comprehensive outcomes, these systems remain limited in implementation. Embracing multimodal data integration offers a promising solution, paving the way for AI-driven healthcare capable of nuanced diagnoses, precise prognostic evaluations, and tailored treatment plans.

The limitations are particularly significant in the fields of radiological image interpretation and clinical decision support systems. Radiologists facing overwhelming image interpretations encounter increased fatigue and higher error rates. Meanwhile, despite proficiency in image analysis, automated systems often struggle to integrate critical clinical context, akin to human physicians’ meticulous approach (Huang et al., 2020a). The importance of integration becomes evident in medical imaging interpretations, where the fusion of heterogeneous data sources including imaging findings, patient demographics, clinical history, and risk factor information is essential.

Furthermore, integrating diverse data modalities in biomedical research has proven beneficial in understanding complex diseases like cancer. For instance, fusing genomic data with histopathological images provides crucial insights into cancer heterogeneity, aiding tailored therapies and improving predictions (M’Sabah et al., 2021; Stahlschmidt, Ulfenborg & Synnergren, 2022). The convergence of various data types consistently demonstrates improved diagnostic accuracy across multiple medical imaging tasks (Huang et al., 2020b; Mammoottil et al., 2022; Sun et al., 2023). The motivation behind utilizing multimodal data in healthcare is its demonstrated ability to substantially enhance diagnostic accuracy, enable personalized treatments, optimize resource allocation, and improve overall healthcare delivery. These advancements promise transformative shifts towards comprehensive healthcare solutions catering to individual patient needs.

In this paper, the terminology ‘data fusion’ refers to the technique of integrating multiple data modalities, while ‘multimodal data’ refers to the combined dataset resulting from this integration. In this study, a fusion of medical healthcare data to form multimodal data using different types of fusion techniques is conducted to collect and synthesize the available literature to establish a foundation for future research. We aim to find all relevant information regarding the fusion techniques of multimodal data and different types of data combinations. In addition, most of the review papers focused on fusion techniques and strategies and surveyed recent trends and advances. In this study, the contributions of this paper are as follows:

1. Our focus extended to analyzing various data fusion techniques in healthcare AI model development. By examining each fusion method, we provided comprehensive insights into the healthcare data fusion landscape, offering valuable guidance for researchers and practitioners.
2. We focused on various multimodal data fusion techniques, including the integration of medical images with structured data, unstructured data, multiple image modalities, and other features. By exploring these techniques, we clarified the strategies applied in healthcare AI model development.
3. We highlighted the applications of multimodal data in various diseases to gain a clear view of the fusion techniques used for specific types of diseases.

In this paper, we have carefully identified and reviewed 69 related works published between 2018 and 2024 that employed data fusion techniques in combining multiple modalities of healthcare data. This paper also provides the links and websites of the public datasets that are normally used by researchers in particular diseases. The paper is organized in the following structure: the Methodology section provides the article selection of this study; the Results section presents data fusion techniques of multimodal data, reviews the papers with related works on data fusion techniques, and discusses different multimodal data in various diseases; and the Discussion section offers a comprehensive discussion of the proposed framework and future works.

## Methodology

### Article selection

**Figure 1:**
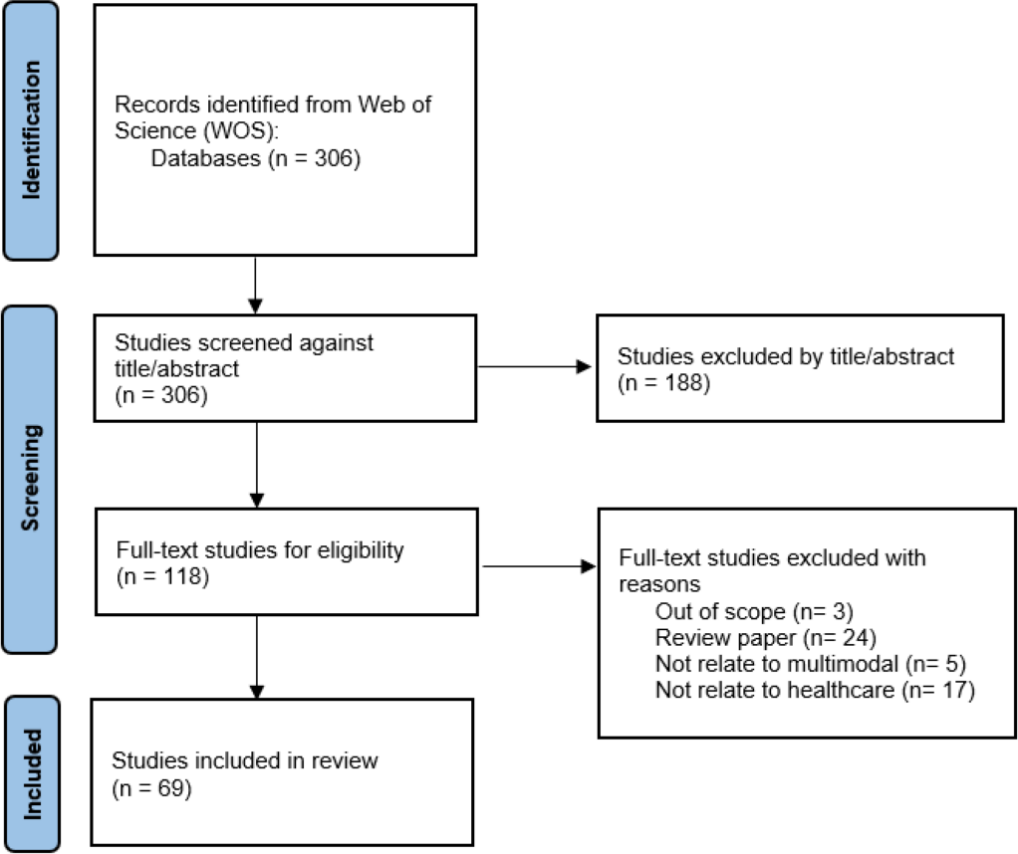
The PRISMA flowchart of article selection process.

**Figure 2:**
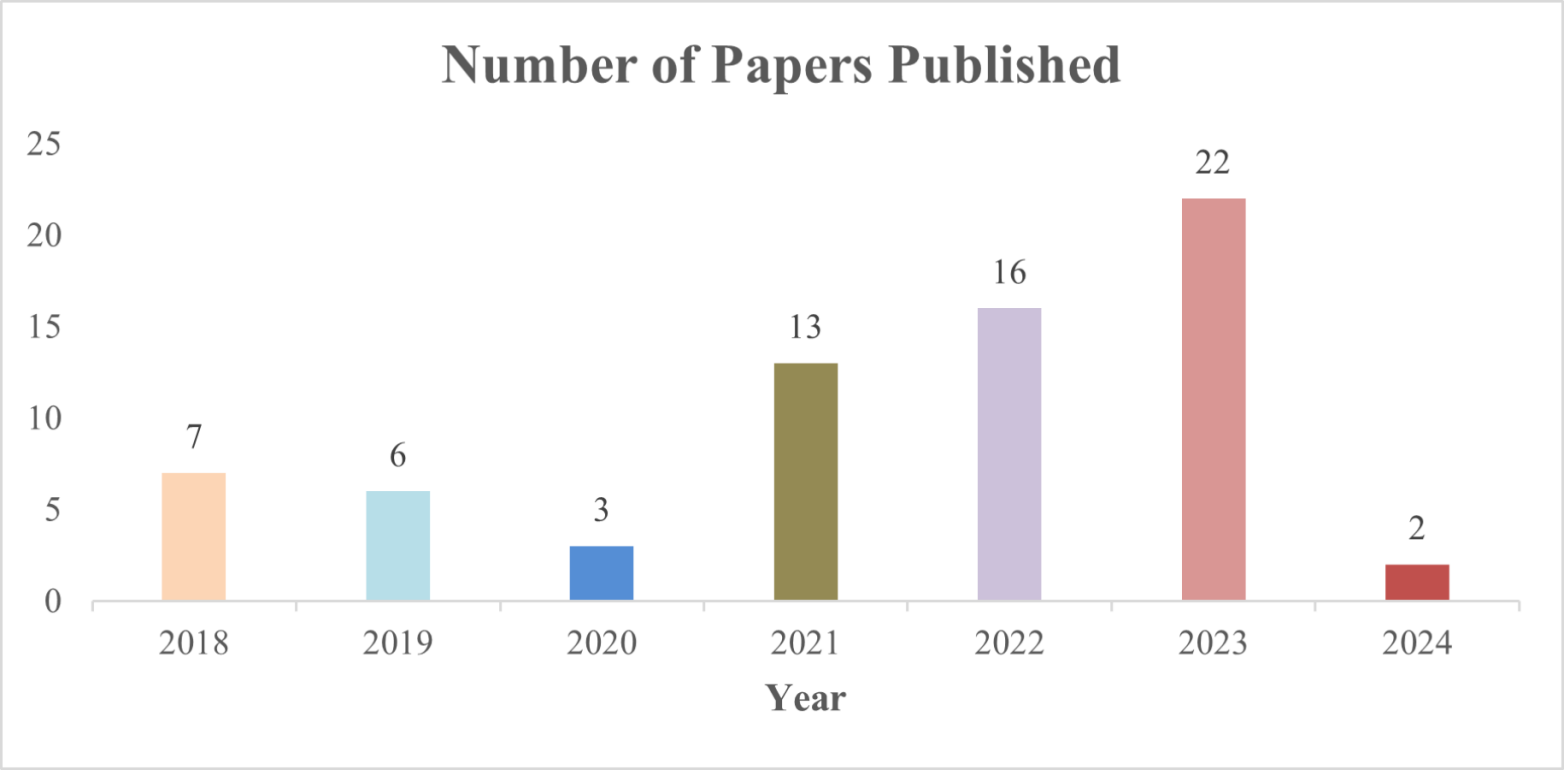
The number of papers published from 2018 to 2024. The increasing research interest in multimodal healthcare data fusion starting from 2021.

In this review paper, we systematically selected relevant studies based on specific inclusion and exclusion criteria to ensure comprehensive coverage and quality, as shown in Fig. 1. The inclusion criteria encompassed papers published from 2018 onwards in the Web of Science (WOS) database, illustrated in Fig. 2. The year 2018 was chosen because it marks the widespread introduction of multimodal data fusion in the healthcare sector. We utilized WOS as our primary resource for finding articles due to its advantages: it is recognized as a reliable and comprehensive database, containing high-quality scholarly journals from various fields, and it implements rigorous quality control measures, such as peer review and citation analysis, to ensure the reliability and credibility of the included literature.

In addition, the following research questions were developed formulated in aiding the process of developing this review:

1. What are the techniques used to fuse multimodal data?
2. Which data can be used to form multimodal data?
3. What are the applications of multimodal data fusion?
4. How does multimodal data benefit the healthcare sector?
5. What types of AI models mostly developed by the researchers using data fusion?
6. What are the challenges and possible solutions towards AI model development using multimodal data fusion?

### Search string

To find relevant articles regarding multimodal data fusion, we used the following search string:

*(“Multimodal Fusion” OR “Multimodal Data Fusion”) AND (“Medical”) AND (“Disease”) AND (“Data Fusion”) AND (“Early Fusion”) AND (“Intermediate Fusion” OR “Joint Fusion”) AND (“Late Fusion”)*

The keywords were chosen to specifically denote the integration of multiple data modalities and ensure the search is confined to the medical field, the primary focus of our study. These keywords help capture studies related to various diseases, making the results pertinent to understanding how multimodal data fusion can aid in disease diagnosis, prognosis, or treatment.

### Study selection criteria

We identified relevant studies using specific selection criteria, considering only articles directly related to the medical field or healthcare sector and employing multimodal data fusion techniques. The title and abstract of each paper were assessed for relevance to our objective of reviewing multimodal data fusion in healthcare. To maintain rigor, we excluded case studies, news items, review papers, and non-English articles. Only articles with full-text access were included to ensure thorough examination and analysis. By adhering to these criteria, we aimed to select high-quality and pertinent studies for our review.

We used VOSviewer to gain insights into the academic landscape of our field. This software visualizes and analyzes bibliometric data. We created co-authorship networks to understand researcher collaboration and analyzed citation networks to identify influential papers and authors. VOSviewer also helped visualize keyword co-occurrences, revealing research trends and clusters.

**Figure 3:**
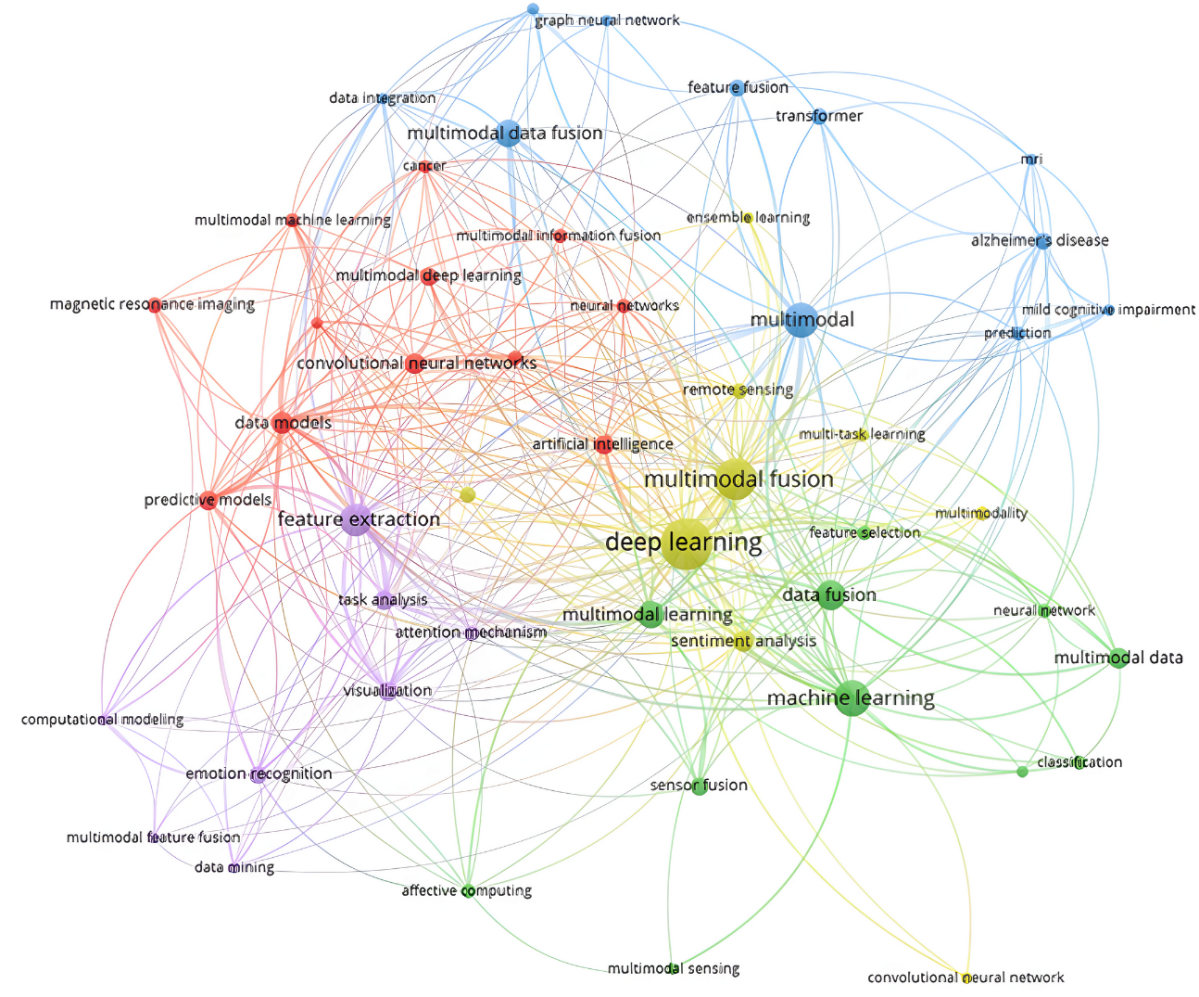
Visual representation of the scientific landscape of the selected studies using VOSviewer’s mapping function.

Visual representation in Fig. 3 highlights the main words of the selected literature, using the abstract, title, and keywords of papers from the Web of Science (WOS) database. Analyzing with VOSviewer revealed 5 clusters (yellow, blue, green, red, and purple), showing relationships between topics. The yellow cluster focuses on deep learning techniques, including multimodal fusion, ensemble learning, multitask learning, and applications like sentiment analysis and remote sensing. The green cluster emphasizes multimodal learning and data fusion, encompassing machine learning techniques, neural networks, and classification algorithms. The purple cluster centers on feature extraction, visualization, and computational modeling, with an emphasis on attention mechanisms and task analysis. The red cluster highlights artificial intelligence applications in cancer research, including predictive models and deep learning approaches. The blue cluster underscores the integration of multimodal data and ensemble learning techniques, focusing on prediction and data fusion strategies. By analyzing the keywords within each cluster, we gain insights into key themes, trends, and research directions, informing further investigation and collaboration.

## Results

### Overview of different fusion techniques for multimodal data for medical applications

**Figure 4:**
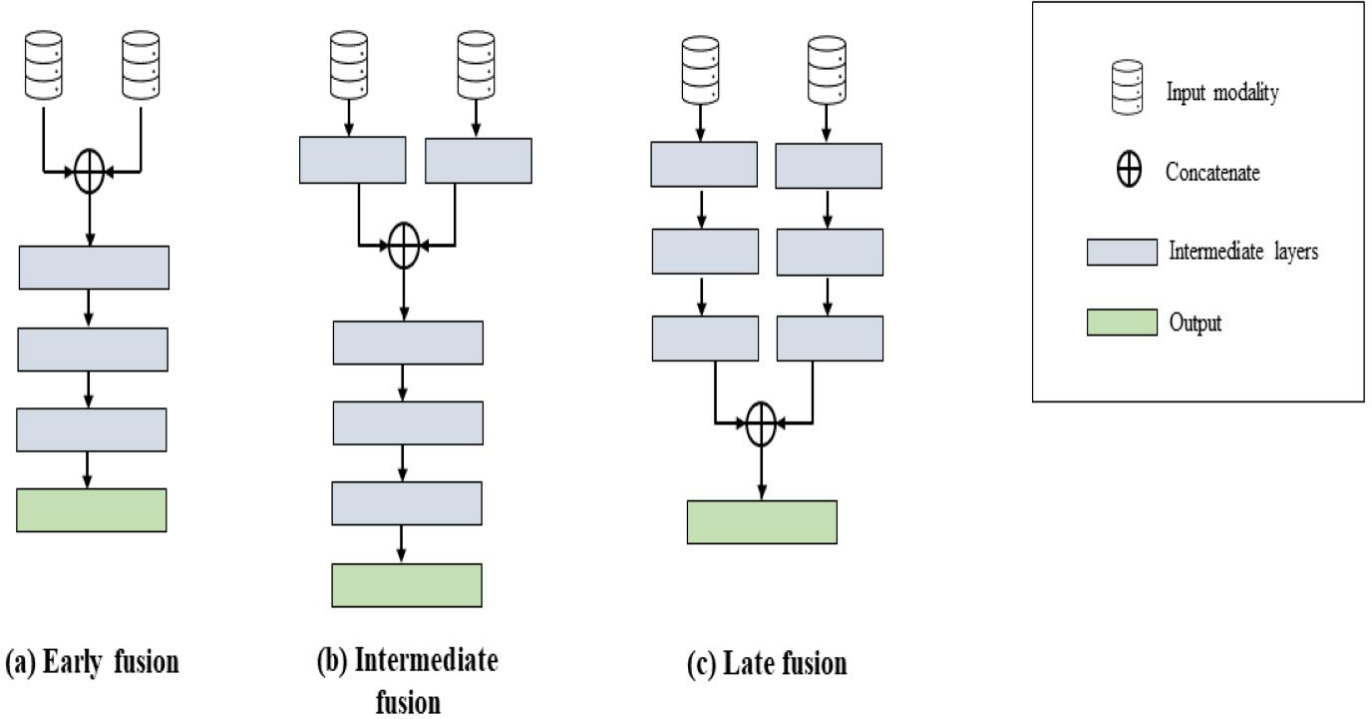
Illustrations of architectures of different fusion techniques.

Data fusion integrates various data types to address inference problems by combining different viewpoints on a phenomenon. This technique leverages features within different data sources to refine estimates and predictions (Mohsen et al., 2022). Combining data from multiple sources, often termed data fusion in biomedical literature, minimizes errors compared to single-source approaches (Stahlschmidt, Ulfenborg & Synnergren, 2022). The primary goal is to extract and integrate complementary contextual information from diverse sources to facilitate decision-making. This approach allows AI models to use information from various sources, particularly beneficial with noisy or incomplete data, enhancing robustness and accuracy (Lipkova et al., 2022). There are three main types of data fusion techniques: early, intermediate or joint, and late fusion, as illustrated in Fig. 4.

Early fusion, also known as feature-level or low-level fusion, consolidates multiple input modalities into a unified feature vector before training a single machine learning model. This process uses methods like concatenation, pooling, or gated units to merge input modalities. There are two primary types: type I combines original features, while type II integrates extracted features from methods such as manual techniques, imaging software, or other neural networks (Huang et al., 2020a; Moshawrab et al., 2023). Early fusion merges modalities based on predictor information or independent variables, serving either as a preprocessing step or an unsupervised task to create features that capture underlying patterns (Gaw, Yousefi & Gahrooei, 2022; Stahlschmidt, Ulfenborg & Synnergren, 2022). While early fusion strategies are effective at learning relationships across modalities from low-level features, they may not capture higher-level relationships that require explicit learning of marginal representations. These strategies can also be sensitive to variations in sampling rates among modalities (Stahlschmidt, Ulfenborg & Synnergren, 2022).

Intermediate fusion, also known as joint or middle fusion, integrates learned feature representations from intermediate layers of neural networks with features from different modalities. Unlike early fusion, intermediate fusion allows the loss during training to influence feature extraction models, refining representations iteratively (Huang et al., 2020a). This approach focuses on learned feature representations rather than original multimodal data, enabling neural networks to learn these representations, whether homogeneously or heterogeneously designed. This can potentially discover more informative latent factors (Stahlschmidt, Ulfenborg & Synnergren, 2022). It is often demonstrated through branched neural network models that merge learned feature representations from intermediate layers with other source features, enhancing the model’s understanding of combined representations (El-Ateif & Idri, 2022; Shetty, Ananthanarayana & Mahale, 2023).

Late fusion, or decision-level fusion, consolidates predictions from multiple models into a final decision. This process involves training separate models for different modalities and then employing an aggregation function to merge these models’ predictions (Huang et al., 2020a). It utilizes different rules, like Max-fusion, Averaged-fusion, or Bayesian rules, to fuse decisions from distinct classifiers (Moshawrab et al., 2023). Late fusion integrates feature vectors from individual modalities *via* separate discriminative models, combining resulting probability values into final feature vectors for each patient. This process incorporates a meta-learner to weigh the significance of each prediction source rather than individual features, thereby enhancing the final label’s accuracy (El-Sappagh et al., 2020).

### Different fusion techniques in healthcare AI model development

**Figure 5:**
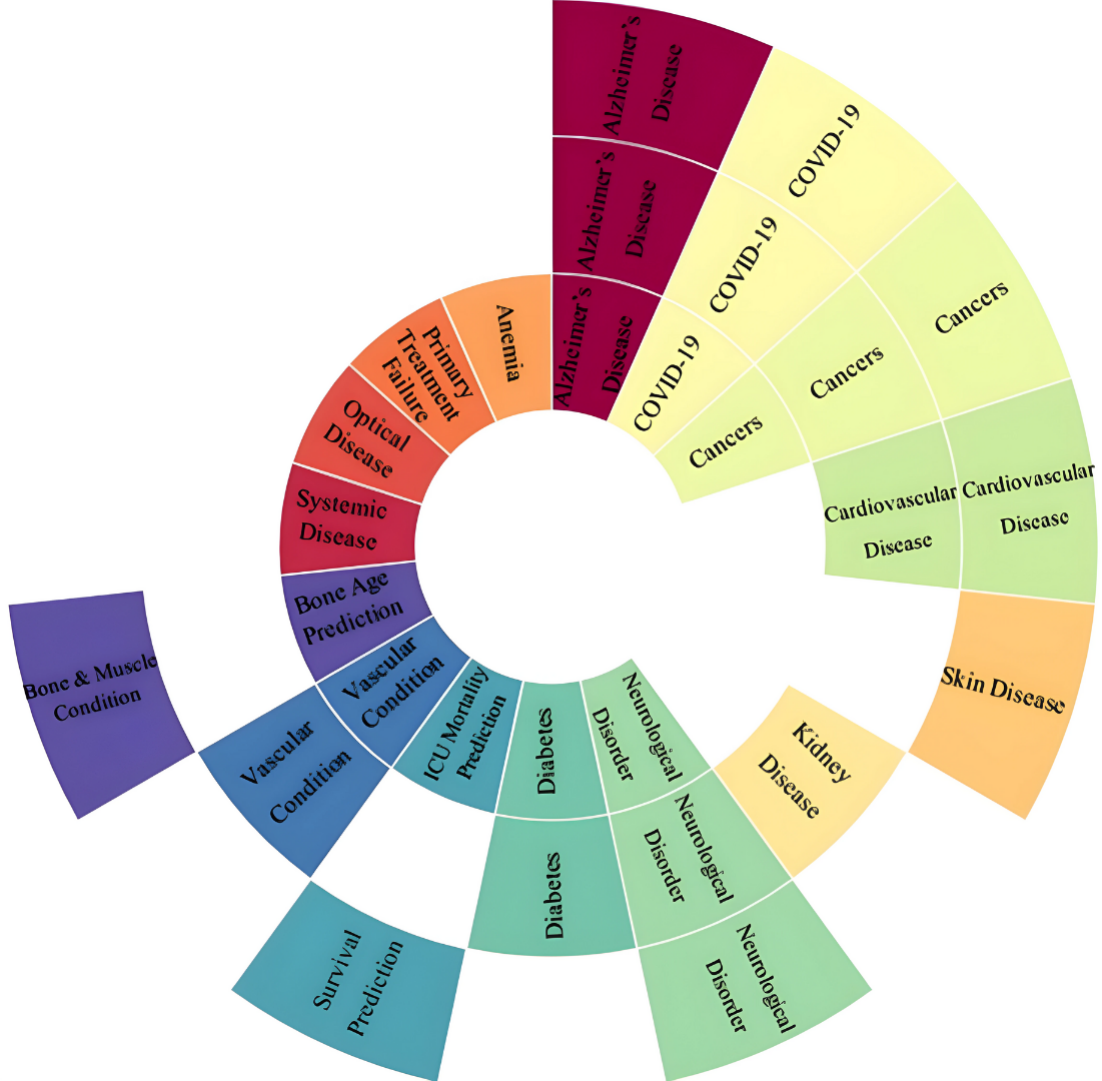
Sunburst chart that represents the types of fusion techniques used in different diseases. Early fusion (inner ring), intermediate or joint fusion (middle ring), and late fusion (outer ring).

The application of early, intermediate, and late fusion in medical condition in various diseases such as Alzheimer’s disease (AD), anemia, various cancer type and many more as shown in Fig. 5. Research on data fusion techniques in the medical field and for various diseases has progressed significantly from 2018 to the present. The highlights and gaps are summarized in Table 1. It is evident from Table 1 that Alzheimer’s disease and various types of cancer have the highest number of published papers. Most studies emphasize the advantages of using multimodal data to develop advanced models for disease detection and prediction. However, some limitations, such as missing data and small sample sizes, are also noted. These limitations and proposed future work are discussed in the Future Trends section.

**Table 1:** Summary table for different types of fusion techniques based on different types of diseases.

**Disease**	**Reference (Author)**	**Early**	**Joint**	**Late**	**Concluding remarks**	**Gap**
Alzheimer	Bhagwat et al. (2018)	/			Most of the technical papers focused on developing prediction and classification models using multimodal data for Alzheimer’s disease. One technical paper focused on developing a detection model. This model aimed to detect the presence of Alzheimer’s disease and mild cognitive impairment. In summary, the technical papers examined in this review collectively highlight the advancements in machine learning and deep learning approaches for Alzheimer’s disease research using data fusion. Some studies achieved significant improvement with approximately 90% accuracy, precision, AUC, recall, and F1-score after employed multimodal data. The consistent use of the ADNI dataset across multiple studies underscores its significance as a valuable resource for Alzheimer’s disease research. There are few studies implemented other datasets for increased and validation of model such as AIBL, PPMI and colorectal cancer dataset.	Challenges include feature selection consistency and missing timepoints.
		Dimitriadis et al. (2018)	/					Limited cohort size for trajectory modeling.
	Li & Fan (2019)	/				
	Dai et al. (2021)	/					
	Chen et al. (2023)	/					
	Li et al. (2023a)	/					
	Odusami et al. (2023)	/					
	Spasov et al. (2018)		/			Inability to consistently predict with 100% accuracy due to outliers.
	Lin et al. (2020)		/			Missing data.
	Abdelaziz, Wang & Elazab (2021)		/			Difficulty in finding ground truth labels for genetic data.
	Golovanevsky, Eickhoff & Singh (2022)		/			
	Rahim et al. (2023b)		/			
	Rahim et al. (2023a)		/			
	Kadri et al. (2023)		/			
	Lu et al. (2024)		/			
	Feng et al. (2019)			/		Limited availability of MRI and PET imaging data.
	Tang et al. (2023)			/		
Anemia	Purwar et al. (2020)	/			The study proposed a method that combines blood smear image features extracted by a deep CNN and clinical features and achieved accuracy, sensitivity, and specificity of 99%, 1.00, and 0.98, respectively.	Limited sample size.
						No comparison with existing diagnostic approaches.
Vascular condition	Liu et al. (2018)	/			The study employed a deep learning approach with multimodal data fusion to develop prediction model. The proposed methods make well prediction. One of the studies achieved overall prediction accuracy of 94.8% and another study predicted severe hemorrhages better than human experts and machine learning models that utilized single data modality.	–
	Akazawa & Hashimoto (2023)		/			MRI image segmentation is not efficient.
						Small sample size.
Covid 19	Kumar et al. (2022a)	/			Most of the studies employed deep learning in multimodal data fusion. Remarkably, most of these studies achieved exceptional accuracy rates of 90% or higher following the integration of multimodal data. This outcome highlights the potential of multimodal data fusion techniques to enhance predictive accuracy and diagnostic capabilities in Covid-19 research. The studies that employed early fusion utilized public datasets in their research and others utilized private datasets.	–
		Kumar et al. (2022b)	/					
	Zhou et al. (2022)		/			Lacks real-world application validation.
	Dipaola et al. (2023)		/			Algorithm efficiency and manual revision requirements for optimization were limitations.
	Xu et al. (2021)			/		Lack of exploration on the effectiveness of clinical data for diagnosis.
	Zheng et al. (2021)			/		Integration challenges between high-dimensional CT imaging and low-dimensional features.
	Zhang et al. (2022)			/		Missing data/ data inadequacy
Cancer	Kharazmi et al. (2018)	/			These studies focused on various cancer types including breast, skin, lung, and prostate, and it is evident that deep-learning methodologies have been extensively utilized for prediction, classification, and detection tasks. These studies focused on various cancer types including breast, skin, lung, and prostate, and deep-learning methodologies have been extensively used for prediction, classification, and detection tasks. Many studies reported accuracy, sensitivity, specificity, and AUC values of 0.80 and above when employing data fusion techniques. This showed the effectiveness of multimodal data fusion in enhancing predictive and diagnostic capabilities.	Limited demographic and tumor-related features used in the model.
		Nie et al. (2019)	/					Small datasets
	Silva & Rohr (2020)	/				Limited clinical information and small number of patients in previous studies.
	Mokni et al. (2021)	/				Sample size imbalance.
	Yan et al. (2021)	/				Potential biases from missing data.
	Joo et al. (2021)	/				
	Tan et al. (2022)	/				
	Oh et al. (2023)	/				
	Yala et al. (2019)		/			Limited sample size.
	Wang et al. (2021)		/			Do not have independent datasets for validation.
	Schulz et al. (2021)		/			Lack of generalization ability of external datasets for model validation.
	Qiu et al. (2022)		/			
	Yao et al. (2022)		/			
	Wei et al. (2023)		/			
	Reda et al. (2018)			/		Lack of validation datasets.
	Fu et al. (2021)			/		Small sample size.
	Yang et al. (2022)			/		
	Caruso et al. (2022)			/		
	Holste et al. (2023)			/		
ICU mortality prediction	Lin et al. (2021)	/			The study proposed deep learning with multimodal data for ICU mortality prediction and the results demonstrated notable improvements in C-index, with values of 0.7847.	Missing value in datasets.
Diabetes	Hsu et al. (2021)	/			The studies focused on multimodal data fusion for diabetes prediction and classification using deep learning techniques. By integrating multiple data modalities, including EHR and imaging data, they achieved notable improvements in accuracy compared to single-modality approaches.	Limited by the availability of EHR data.
	Hu et al. (2023)	/				
	El-Ateif & Idri (2022)		/			–
Diffuse large B-cell lymphoma (DLBCL)	Yuan et al. (2023)	/			This study constructed multimodal deep learning by integrating multiple image modalities and EHR. It achieved 91.22% and 0.925 of accuracy and AUC after optimization of model.	–
Optical disease	Chaganti et al. (2019)	/			Both studies employed CNN for prediction and detection task using early fusion. The merged CNN for the first study achieved AUC of 0.74 while another study achieved AUC of 0.9796. However, both studies showed improvement after implementing multimodal data.	The study lacks direct segmentation for optic nerve volume estimation.
	Jin et al. (2022)	/				
Neurological disorder	Zhang et al. (2023)	/			These studies worked on multimodal deep learning by integrating MRI and clinical data. The results showed significant improvements in AUC and accuracy.	Data insufficiency or model simplicity.
		Yoo et al. (2019)		/				Limited training samples.
	Huang et al. (2022)			/		–
Bone and muscle	Li et al. (2023b)	/			The authors reported that multimodal deep learning models outperformed the traditional approach with improved accuracy, sensitivity, and AUC.	–
	Jujjavarapu et al. (2023)			/		Deep learning is computationally expensive and less interpretable.
	Schilcher et al. (2024)			/		
Systemic disease	Zhao et al. (2022)	/			The author developed a multimodal deep learning method to predict systemic diseases using oral condition. The best accuracy and AUC achieved by the model are 0.92 and 0.88 respectively.	Limited generalizability.
Cardiovascular disease	Yao et al. (2023)		/		The studies highlighted that the use of multimodal deep learning architectures demonstrates superior performance compared to unimodal approaches, showcasing the importance of integrating multiple healthcare data such as chest X-ray images, fundus images, ECG data and EHR. The significant improvements shown in the high accuracy and AUC of models were achieved.	Small dataset.
	Lee et al. (2023)		/			
	Hsieh et al. (2023)		/				
	Puyol-Anton et al. (2022)			/		Lack of testing with the same pipeline for predicting response to other types of treatment.
	Jacenków, O’Neil & Tsaftaris (2022)			/		
Kidney disease	Chen et al. (2023)		/		The author reported that fusion technique improved sensitivity (0.822) in detecting hyperplastic parathyroid glands for chronic kidney disease.	Lack of spatial information.
						False-positive results.
Survival prediction	Chen et al. (2021)			/	The proposed method consistently outperformed state-of-the-art methods in survival outcome prediction in computational pathology, achieving superior performance with a 3.0% to 6.87% in overall C-Index.	Using previously curated gene set with potentially overlapping biological functional impact.
Skin disease	Cai et al. (2023)	/			Both studies proposed multimodal deep learning classification model that outperformed a baseline method. They combined multiple imaging modalities such as dermatoscopic images and macroscopic images with patient metadata for skin lesion classification.	–
	Yap, Yolland & Tschandl (2018)			/		No comparison with human physicians limits clinical relevance insights.

### Early fusion

A total of 69 studies were identified in this research endeavor. The predominant utilization of early fusion (28/69) and intermediate fusion (23/69) methodologies was observed in integrating multimodal healthcare data, with a comparatively lesser emphasis on late fusion (18/69). Among these twenty-eight early fusion studies, seven focused on diagnosing and predicting Alzheimer’s disease, while six were dedicated to cancer classification, with the remaining studies addressing other diseases.

For instance, among seven studies in AD, there are two significant studies which employed longitudinal data for disease predictions. Bhagwat et al. (2018) focused on the prediction of clinical symptoms trajectories of AD by training a Longitudinal Siamese Neural Network (LSN) on longitudinal multimodal data. The author successfully applied cross-validation using three different ADNI cohorts and achieved generalizability on validation dataset of AIBL dataset. The LSN achieved 0.900 accuracy and 0.968 AUC on ADNI datasets and achieved 0.724 accuracy and 0.883 AUC on the replication AIBL dataset. The study showed potential improvement in prognostic predictions and patient care in AD.

Various cancer types, such as breast cancer, lung cancer, skin cancer, and brain tumors, have employed multimodal data for AI development. For example, Yan et al. (2021) proposed deep learning architectures such as CNN and VGG-16 for breast cancer classification based on multimodal data. The author employed denoising autoencoder to increase low-dimensional structured EHRs data to high-dimensional so that it can be fed into the CNN with pathological images. The proposed method improved breast cancer classification accuracy up to 92.9%.

### Intermediate fusion

There are 23 studies related to multimodal fusion utilizing healthcare data. Among these, a predominant focus has been on Alzheimer’s disease, exploring prediction models utilizing deep learning architectures. Lin et al. (2020) employed Extreme Learning Machine (ELM) with multiple modalities fusion to predict AD conversion within 3 years. In feature selection process, the author utilized the least absolute shrinkage and selection operator (LASSO) algorithm which had been said to be beneficial in selecting MRI features. Thus, the proposed model achieved 87.1% accuracy and AUC of 94.7 in predicting AD conversion.

In another study, Akazawa & Hashimoto (2023) employed pretrained VGG-16 for MRI image, alongside CNN for extracting features from both laboratory and demographic data. These features were then concatenated using a neural network to develop a prediction model for severe hemorrhage, surpassing the performance of human experts and single data type models.

Additionally, Yoo et al. (2019) implemented multimodal deep learning method in predicting multiple sclerosis conversion. The author combined user-defined MRI and clinical measurement in their proposed model and employed a technique called Euclidean distance transform to increase information density in multiple sclerosis lesion masks. The CNN-based prediction model achieved 75.0% accuracy in predicting disease activity within two years and outperformed random forest model that only used user-defined measurements.

### Late fusion

In this section, a total of 18 studies have leveraged late fusion techniques in multimodal data fusion to develop detection and prediction models for various diseases. Notably, Feng et al. (2019) employed late fusion by incorporating primary features extracted from MRI and PET images into 3D-CNN deep learning architectures. Besides, the author applied FSBi-LSTM on hidden spatial information to enhance performance of model, resulting in enhanced diagnostic accuracy of 94.82%.

For prostate cancer diagnosis, Reda et al. (2018) employed late fusion techniques by concatenating outputs from diverse classifiers, integrating clinical biomarkers and extracted features from diffusion-weighted magnetic resonance imaging (DW-MRI). The early diagnosis system achieved 94.4% diagnosis accuracy with 88.9% sensitivity and 100% specificity on 18 DW-MRI datasets, indicating promising results for the computer-aided diagnostic system.

In the cardiovascular field, Puyol-Anton et al. (2022) developed a multimodal deep learning framework (MMDL) by using 2D Deep Canonical Correlation Analysis (DCCA) algorithm for Cardiac resynchronization therapy (CRT) response prediction. By combining multimodal data, they achieved a CRT response prediction accuracy of 77.38%, demonstrating that the MMDL classifier improves accuracy compared to baseline approaches. Overall, late fusion techniques have shown efficacy in enhancing disease detection and prediction models across diverse medical domains.

### Type of multimodal data

**Figure 6:**
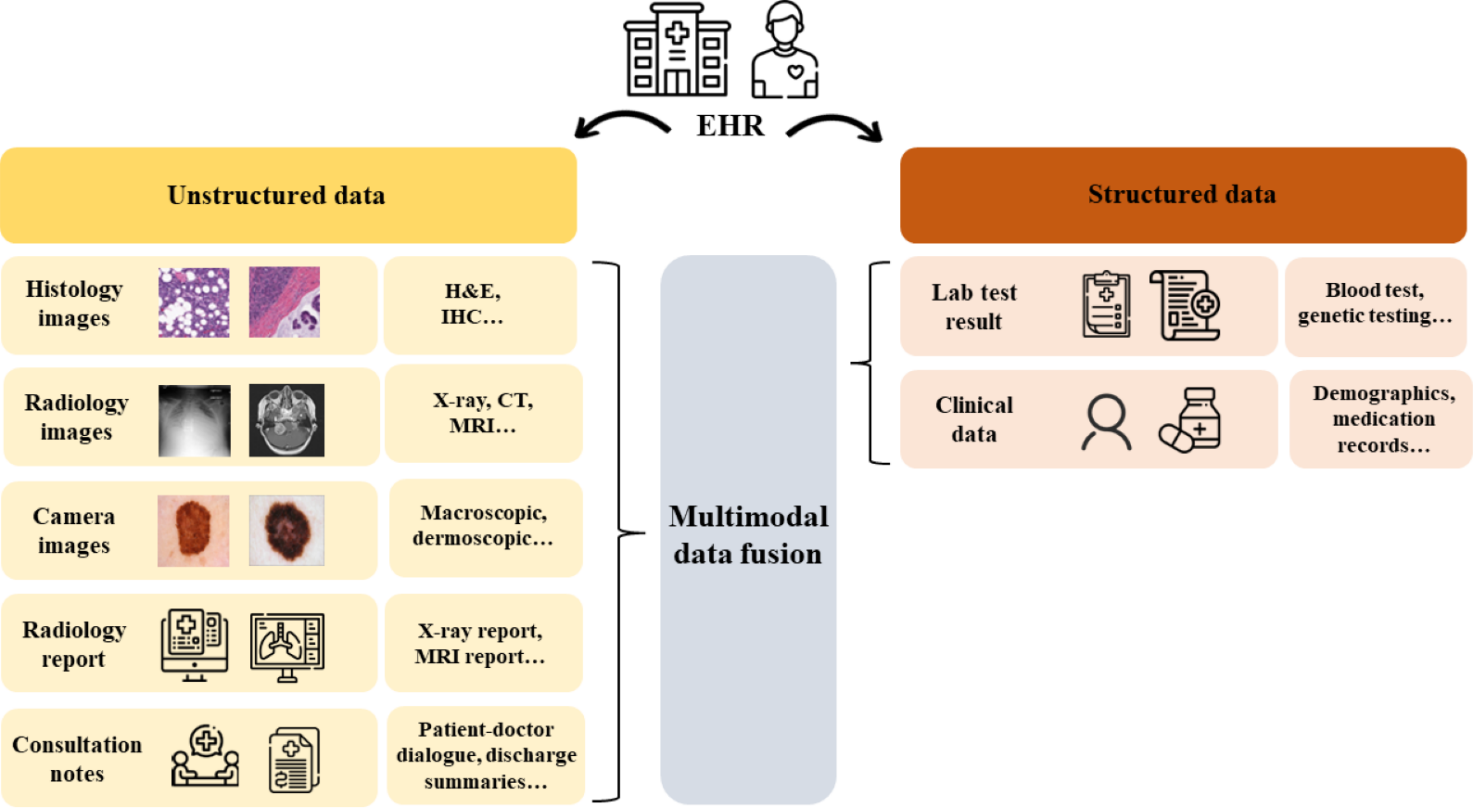
Example types of unstructured and structured data in EHR. Image sources: Hospital icon: https://www.freepik.com/icon/hospital_4320350; Patient icon: https://www.flaticon.com/free-icon/patient_1430402; Medical history icon: https://www.freepik.com/icon/medical-history_1424569; X-ray icon: https://www.freepik.com/icon/x-ray_7399390; Consultation icon: https://www.flaticon.com/free-icon/consultation_10202726?; Medical report icon: https://www.flaticon.com/free-icon/medical-report_3215528?; Medical checkup icon: https://www.flaticon.com/free-icon/medical-checkup_3061457?; Medicine icon: https://www.freepik.com/icon/medicine_4063711; People icon: https://www.veryicon.com/icons/miscellaneous/8atour/people-23.html; Medicine icon: https://www.freepik.com/icon/medicine_4063711; Lab result icon: https://www.flaticon.com/free-icon/invoice_751904; Histological images: https://www.kaggle.com/datasets/paultimothymooney/breast-histopathology-images/data; camera images (skin lesion): Tschandl P, Rosendahl C & Kittler H. The HAM10000 dataset, a large collection of multi-source dermatoscopic images of common pigmented skin lesions. Sci. Data 5: 180161 DOI: 10.1038/sdata.2018.161 (2018); X ray image: https://www.kaggle.com/datasets/financekim/curated-cxr-report-generation-dataset, Public Domain; brain MRI: https://www.kaggle.com/datasets/masoudnickparvar/brain-tumor-mri-dataset.

Health data categorizes into three main types: imaging, clinical, and omics data. Each category provides unique insights, but their fusion yields a fuller disease comprehension, reducing ambiguity and enhancing model efficiency in medical data analysis. Imaging methods like MRI, CT, PET, and SPECT offer varied perspectives on anatomy and physiology. Clinical data, including patient histories, age, gender, and medication records, aid clinicians in understanding patient characteristics and disease progression, enhancing the contextual understanding of patient health. Additionally, genetic data play a crucial role in predicting and diagnosing conditions, providing valuable insights into disease progression and individualized treatment (Behrad & Abadeh, 2022). The example types of unstructured and structured data are shown in Fig. 6.

### Fusion of image and structured data

**Figure 7:**
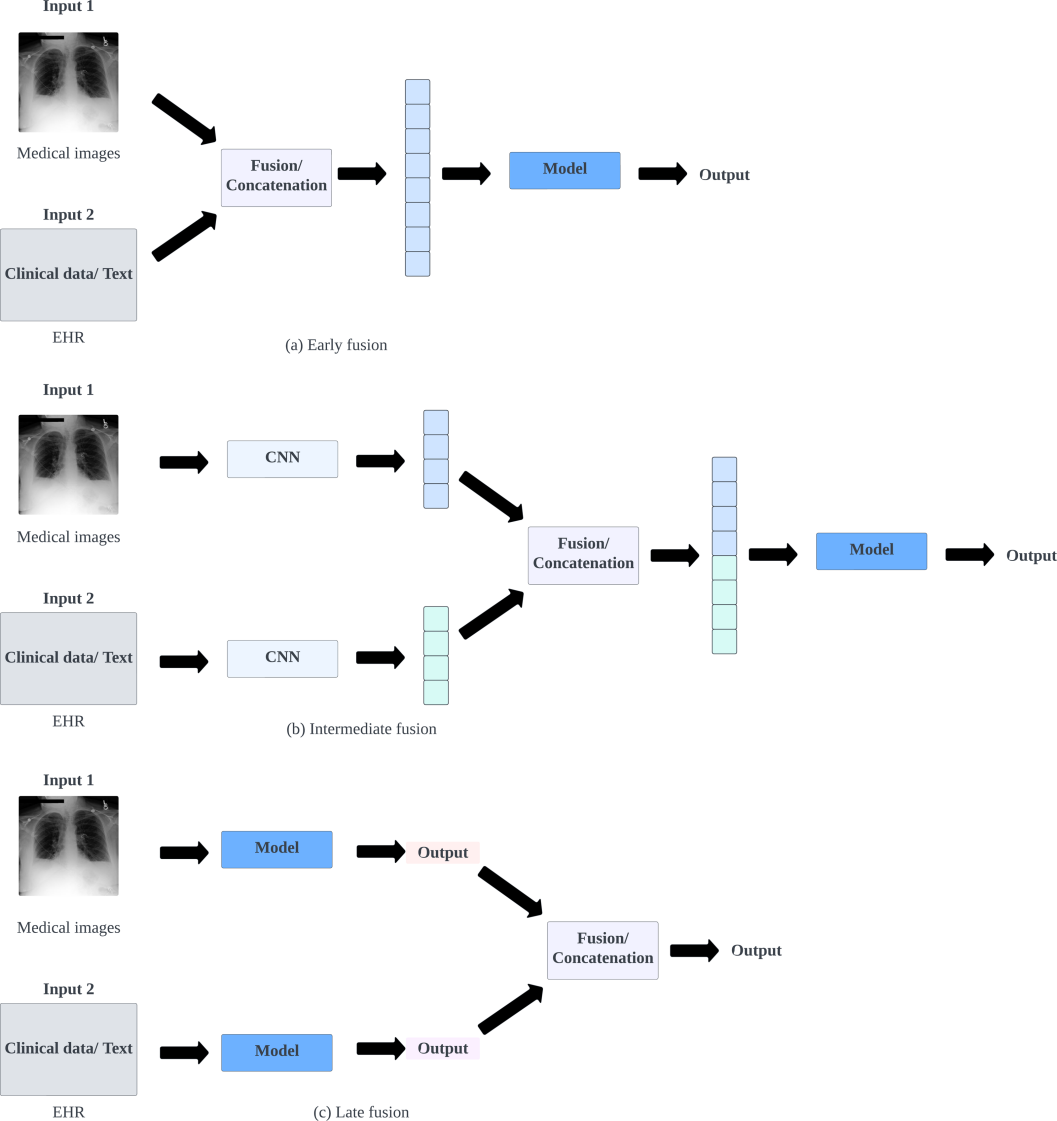
Type of data fusion framework for image and structure data using early, intermediate, and late fusion. X ray images: https://www.kaggle.com/datasets/financekim/curated-cxr-report-generation-dataset, Public Domain.

The integration of medical images and EHR has emerged as a pivotal strategy in enhancing predictive modeling across diverse medical domains. An example of architectures in fusing medical images and structured data from EHR is shown in Fig. 7.

In recent years, there has been a growing body of research focused on utilizing medical images and EHRs in Alzheimer’s disease research. In eight studies, Bhagwat et al. (2018), Spasov et al. (2018), Dimitriadis et al. (2018), Li & Fan (2019), Golovanevsky, Eickhoff & Singh (2022), Rahim et al. (2023b); Rahim et al. (2023a) and Lu et al. (2024), they employed MRI in conjunction with clinical assessments, demographic details, and genetic data, particularly the APOe4 marker, from the Alzheimer’s Disease Neuroimaging Initiative (ADNI) dataset. These technical papers have significantly contributed to the understanding and diagnosis of AD, as well as to the development of potential treatment strategies.

In the context of anemia, Purwar et al. (2020) undertook an innovative approach in anemia detection and prediction, integrating blood smear images with clinical data from complete blood count test (CBC) from AIIMS datasets. The features are extracted by deep CNN and fusion technique is applied. The dimensions of fused datasets are reduced by using linear discriminant analysis (LDA) and principal component analysis (PCA). The resulting model exhibited remarkable accuracy, reaching a maximum of 99%.

In addition, Puyol-Anton et al. (2022) explored cardiovascular magnetic resonance images (CMR) and electrocardiogram (ECG) data from UK Biobank and EchoNet-Dynamic to predict the response to cardiac resynchronization therapy (CRT) for heart failure patients. The combination of medical images and healthcare data enables CRT response prediction with 77.38% accuracy which is comparable with the current state-of-the-art in machine learning-based CRT response prediction.

### Fusion of image and unstructured data

In Covid-19, Kumar et al. (2022a); Kumar et al. (2022b) conducted pioneering research by exploring healthcare data fusion techniques involving chest X-ray images and audio data of cough for early diagnosis and accurate classification of Covid-19 cases. They utilized several public datasets such as IEEE-8023 CXR—Cohen dataset, Coswara, Coughvid and many more as stated in Table 2. Additionally, Zheng et al. (2021) employed a comprehensive approach by fusing various image modalities, including X-ray, CT scan, and ultrasound images, with medical consultation data to enhance the classification of COVID-19 cases.

**Table 2:** Summary table of public datasets used by each study.

**Disease**	**Type of data**	**Dataset used**	**Reference**
Alzheimer disease	PET, CT, MRI, Age, gender, education years, APOE *ɛ*4 status at baseline, cerebrospinal fluid biomarkers, gene data, cognitive scores, SNP	ADNI https://adni.loni.usc.edu/data-samples/access-data/	Bhagwat et al. (2018)
					Dimitriadis et al. (2018)
					Li & Fan (2019)
			Dai et al. (2021)
			Chen et al. (2023)
			Li et al. (2023a)
			Odusami et al. (2023)
			Spasov et al. (2018)
			Lin et al. (2020)
			Abdelaziz, Wang & Elazab (2021)
			Golovanevsky, Eickhoff & Singh (2022)
			Rahim et al. (2023b)
			Rahim et al. (2023a)
			Kadri et al. (2023)
			Lu et al. (2024)
		OASIS https://www.oasis-brains.org/	Kadri et al. (2023)
		PPMI https://www.ppmi-info.org/access-data-specimens/data	Dai et al. (2021)
		AIBL http://adni.loni.usc.edu/category/aibl-study-data/	Bhagwat et al. (2018)
Bone age assessment	X-rays (key bone regions), gender	RSNA dataset https://www.rsna.org/rsnai/ai-image-challenge/RSNA-Pediatric-Bone-Age-Challenge-2017	Li et al. (2023b)
Microcytic hypochromia (Anemia)	Blood smear image, clinical features	AIIMS https://www.bioailab.org/datasets	Purwar et al. (2020)
Cancer prediction	Microscopy slides, Clinical data (cancer type, gender, race, history of prior malignancy, and age)	TGCA https://portal.gdc.cancer.gov/	Silva & Rohr (2020)
Survival outcome prediction	Whole slides images, genomic data			Chen et al. (2021)
Prediction of HER2-positive breast cancer recurrence and metastasis risk	Whole slide H&E images (WSIs) and clinical information			Yang et al. (2022)
Colorectal cancer	Pathological images, multi-omic data		Qiu et al. (2022)
Renal cancer	Histopathological images, CT/MRI scans, and genomic data from whole exome sequencing	KIRC TCGA (Kidney renal clear cell carcinoma of the Cancer Genome Atlas) GDC portal https://portal.gdc.cancer.gov/ cancer imaging archive https://www.cancerimagingarchive.net/	Schulz et al. (2021)
Covid	Chest X-ray and cough sample data	IEEE-8023 CXR—Cohen dataset https://github.com/ieee8023/covid-chestxray-dataset Shenzhen CXR with Masks https://www.kaggle.com/datasets/yoctoman/shcxr-lung-mask Montgomery county CXR images https://paperswithcode.com/dataset/montgomery-county-x-ray-set COVIDGR 1.0 https://paperswithcode.com/dataset/covidgr	Kumar et al. (2022a)
		Coswara https://github.com/iiscleap/Coswara-Data Coughvid https://cs.paperswithcode.com/paper/the-coughvid-crowdsourcing-dataset-a-corpus DetectNow https://github.com/shresthagrawal/detect-now Virufy https://github.com/virufy/virufy-data	Kumar et al. (2022b)
ICU-mortality prediction	Chest X-ray, clinical data (EHR), radiology reports	MIMIC IV https://physionet.org/content/mimiciv/2.2/	Lin et al. (2021)
Diabetes	Fundus and WGBF	APTOS 2019 blindness detection https://www.kaggle.com/c/aptos2019-blindness-detection/data Messidor-2 https://www.kaggle.com/datasets/mariaherrerot/messidor2preprocess/data	El-Ateif & Idri (2022)
Breast cancer	Pathological images, EMR	PathoEMR dataset no longer available	Yan et al. (2021)
	Whole-slide images and gene expression profiles	TGCA https://portal.gdc.cancer.gov/	Wang et al. (2021)
Cardiovascular disease	Chest X-rays, report	MIMIC CXR https://physionet.org/content/mimic-cxr/2.0.0/	Jacenków, O’Neil & Tsaftaris (2022)
	Clinical risk factors and fundus photographs	UK Biobank https://www.ukbiobank.ac.uk/enable-your-research/apply-for-access	Lee et al. (2023)
Cardiac resynchronization therapy response prediction	CMR imaging, ECG data	UK Biobank (UKBB) https://www.ukbiobank.ac.uk/enable-your-research/apply-for-access EchoNet-Dynamic https://echonet.github.io/dynamic/	Puyol-Anton et al. (2022)
Disease location in chest X-ray images	Chest X-ray, clinical data	MIMIC-CXR https://physionet.org/content/mimic-cxr/2.0.0/ MIMIC IV https://physionet.org/content/mimiciv/2.2/ REFLACX https://paperswithcode.com/dataset/reflacx	Hsieh et al. (2023)
Prostate cancer	MRI, clinical biomarkers	DW-MRI https://data.mendeley.com/datasets/fgf86jdfg6/1	Reda et al. (2018)
Lung cancer	CT image, lung tumor biomarker	LCID https://wiki.cancerimagingarchive.net/pages/viewpage.action?pageId=1966254	Fu et al. (2021)
	CT, clinical data	CLARO https://paperswithcode.com/dataset/claro	Caruso et al. (2022)

Jacenków, O’Neil & Tsaftaris (2022) demonstrated an innovative application of data fusion by combining chest X-ray images with their corresponding radiological reports to develop a classification model for cardiovascular diseases. This approach integrates radiological images with textual information from MIMIC CXR, enriching the diagnostic process for cardiovascular conditions. By integrating different modalities, the proposed method achieved an average micro AUROC of 87.8, outperforming the state-of-the-art methods for unimodal of 84.4 AUROC.

### Fusion of multiple types of images

Several studies have explored image fusion techniques for Alzheimer’s disease detection. Noteworthy contributions include the work of Dai et al. (2021), which advocate for merging Positron Emission Tomography (PET) with Magnetic Resonance Imaging (MRI) from ADNI and PPMI datasets. The paper proposed a classification model for Alzheimer’s disease diagnosis based on improved CNN models and image fusion method, achieving high AUC values of 0.941 in training with fusion images. The research demonstrated that the proposed method using fusion images dataset based on multi-modality images has higher diagnosis accuracy than single modality images dataset. Meanwhile, Kadri et al. (2023) further extended the exploration by combining MRI with both PET and CT, showcasing the versatility of data fusion in Alzheimer’s disease diagnosis.

Within the domain of cancer research, Mokni et al. (2021) conducted a notable study wherein they employed data fusion techniques. Specifically, they integrated Dynamic Contrast-Enhanced Magnetic Resonance Imaging (DCE-MRI) with Digital Mammographic images (MGs) for the purpose of detecting breast cancer. The author extracted the features by using Gradient Local Information Pattern (GLIP) and performed Canonical Correlation Analysis (CCA) for multimodal fusion. The proposed method achieved an AUC value of 99.10% compared to AUC values for MG and DCE-MRI modalities alone of 97.20% and 93.50%, respectively. This integrative approach capitalizes on the complementary strengths of DCE-MRI and MGs, offering a more comprehensive and detailed insight into breast cancer characteristics, ultimately contributing to improved diagnostic accuracy.

### Other fusion features

In a significant contribution to the field of vascular conditions, Liu et al. (2018) conducted a comprehensive study focusing on the prediction of anterior communicating artery (ACOM) aneurysms. The research involved the integration of diverse healthcare data, including CT images, EHR, and textual reports. By combining these various sources of information, the study aimed to enhance the accuracy and depth of predicting ACOM aneurysms, illustrating the potential of data fusion in advancing vascular condition diagnostics.

Lin et al. (2021) made notable strides in predicting mortality rates in Intensive Care Units (ICUs) by exploring different healthcare data sources. The study integrated chest X-ray images, clinical data, and radiological reports from MIMIC IV to develop a robust model for predicting mortality in the ICU. The contributions of labels, text, and image features are demonstrated as shown in the C-index of the model achieved which is 0.7847, surpassing the baseline model.

Zhang et al. (2023) made significant advancements in the detection of multiple sclerosis (MS), a neurological disorder by involving the fusion of various data sources, including brain MRI images, EHR, and free-text reports from patients’ clinical notes. The proposed method successfully predicts MS severity with an increase of 19% AUROC. This comprehensive fusion of structured and unstructured data enables a more accurate prediction of multiple sclerosis, showcasing the potential of data integration in advancing neurological disorder prediction

## Discussion

In the previous sections, a comprehensive review of recent studies from 2018 to the present focused on machine learning and deep learning techniques for diagnosing, prognosing, and predicting treatments for various diseases. The data fusion combinations are categorized into fusion of medical images with structured data, fusion of medical images with unstructured data, fusion of multiple image modalities, and other features fusion. Additionally, the data fusion techniques were classified into early, intermediate, and late fusion approaches.

Our analysis revealed that multimodal data fusion models consistently outperformed single-modality models across performance metrics such as accuracy, sensitivity, precision, AUC, and C-index. Therefore, it is recommended to employ a multimodal machine learning or deep learning model when multiple healthcare data sources are available, as incorporating additional clinical data from EHR often results in improved performance.

**Figure 8:**
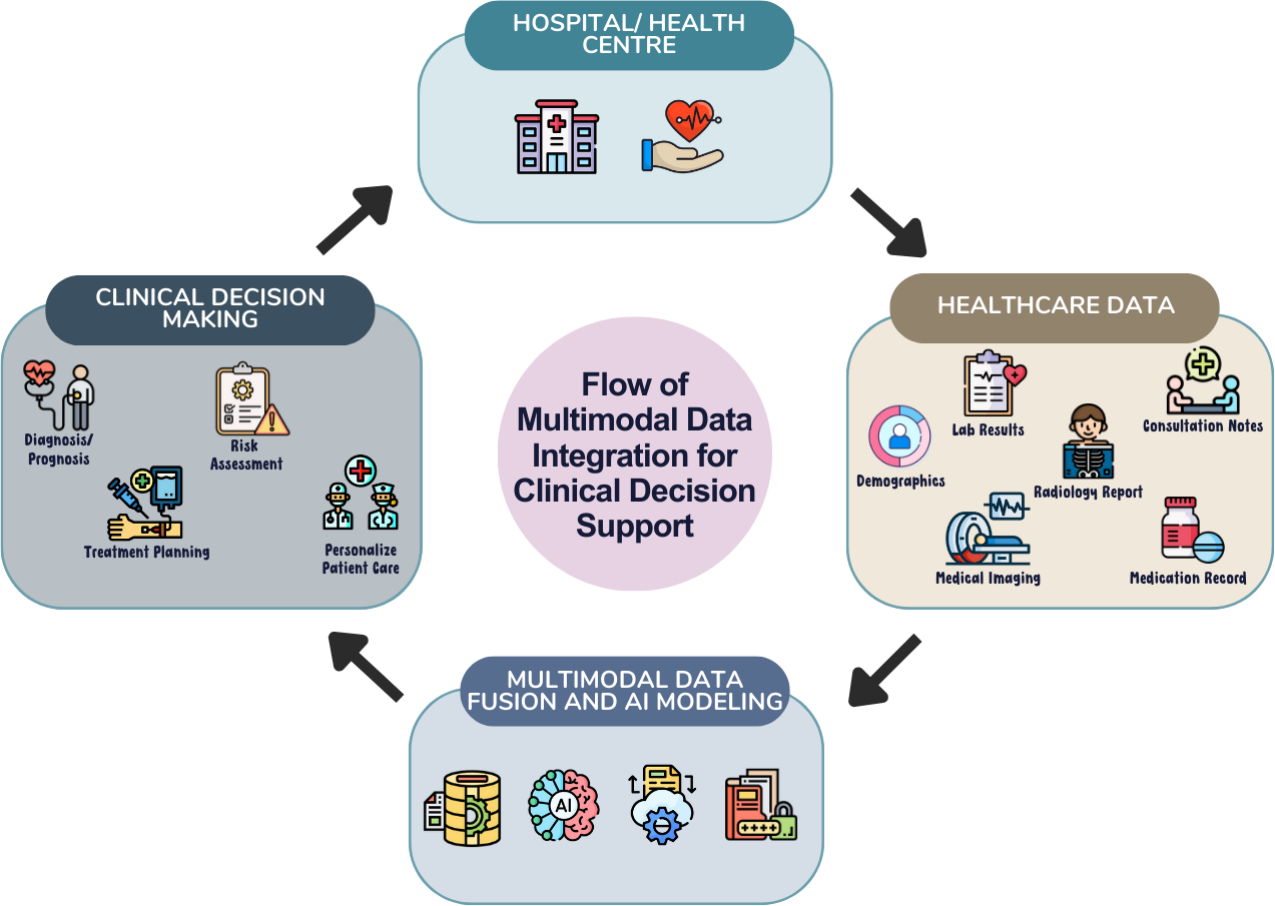
Proposed flow of multimodal data integration for clinical decision support. Image sources: Hospital icon: https://www.freepik.com/icon/hospital_4320350; Cardiogram icon: https://www.flaticon.com/free-icon/cardiogram_7918446; Demographics icon: https://www.freepik.com/icon/demographics_2720724; CT scan icon: https://www.flaticon.com/free-icon/ct-scan_2355587; Aspirin icon: https://www.freepik.com/icon/aspirin_4320310; X-rays icon: https://www.flaticon.com/free-icon/x-rays_706196; Consultation icon: https://www.flaticon.com/free-icon/consultation_10202688; Diagnosis icon: https://www.freepik.com/icon/diagnosis_4320491; Risk management icon: https://www.flaticon.com/free-icon/risk-management_10240181; Diagnosis icon: https://www.flaticon.com/free-icon/diagnosis_1934430; Treatment icon: https://www.freepik.com/icon/treatment_3027624; Medical staff icon: https://www.flaticon.com/free-icon/medical-staff_6190390; Data processing icon: https://www.flaticon.com/free-icon/data-processing_2980479; AI icon: https://www.flaticon.com/free-icon/ai_8131880; Data synchronization icon: https://www.flaticon.com/free-icon/data-synchronization_4403186; Data encryption icon: https://www.flaticon.com/free-icon/data-encryption_4736094.

Figure 8 shows the proposed framework of this study for improving clinical decision support using multimodal data integration. The framework follows a cyclical pattern that begins with the collection of data from various hospitals or health centers. This data is then aggregated through multimodal data fusion and undergoes AI modeling processes. The algorithms analyze the data to extract valuable insights related to health outcomes, including diagnosis, prognosis, risk assessment, and treatment planning. These insights are communicated back to hospitals and practitioners, enabling informed decisions for patients.

The proposed framework for multimodal data integration in clinical decision support offers promising solutions to address several challenges faced by the current healthcare sector. One significant challenge lies in the limited availability of healthcare data such as medical images, clinical data and EHRs (Feng et al., 2019; Hsu et al., 2021; Nie et al., 2019; Zhang et al., 2023). By incorporating advanced data integration techniques, the proposed framework enables the integration of diverse types of healthcare data sources, thereby enhancing access to comprehensive and longitudinal patient health records. This facilitates more accurate diagnoses, enabling more informed clinical decision-making and personalized treatment strategies.

The lack of real-world application and the absence of comparison with human physicians in current healthcare practices represent another critical challenge that the proposed framework seeks to overcome (Xu et al., 2021; Yap, Yolland & Tschandl, 2018; Zhou et al., 2022). The proposed framework addresses this limitation by enabling the integration of expert knowledge and clinical guidelines into decision support systems, thereby facilitating comparative analyses between algorithmic predictions and human expert judgments. This not only enhances the interpretability and trustworthiness of algorithmic recommendations but also encourages collaboration between clinicians and data scientists in optimizing clinical decision-making processes.

### Future trends

Future work aimed at addressing the research gap identified in related studies should prioritize several key areas to enhance the field of healthcare data fusion and multimodal deep learning for clinical decision support. These include dealing with noisy or irrelevant data that may impact model performance, as well as addressing issues related to missing or sparse data (Bhagwat et al., 2018; Lin et al., 2021; Spasov et al., 2018; Tan et al., 2022; Zheng et al., 2021). To tackle this, future research efforts should incorporate robust data imputation methods to address missing data issues effectively. Basic imputation techniques like k-nearest neighbors (KNN) can provide a foundation, while more advanced methods such as matrix completion and deep learning-based approaches can be explored to accurately estimate missing values and improve the quality of input data.

Besides, a significant barrier to the clinical implementation of multimodal deep learning methods is the limited availability of data (Feng et al., 2019; Hsu et al., 2021; Joo et al., 2021; Nie et al., 2019; Zhang et al., 2023). To address this issue, future researchers can implement data synthesis models that can learn the underlying data distribution and generate realistic data samples. The example of the models are generative adversarial networks (GANs) or variational autoencoders (VAEs). However, it is important to note that GANs and VAEs might produce augmented data that significantly differs from the raw data, potentially affecting model performance. When there is limited labeled data, semi-supervised learning is suggested. By exploring semi-supervised learning, models can be trained with both labeled and unlabeled data, effectively utilizing limited labeled data and unlabeled data to improve model performance.

Not only this, enhancing data sharing practices and improving access to comprehensive datasets are crucial steps toward advancing research in this field. Collaborative data-sharing platforms and standardized data collection protocols can help mitigate this challenge. By fostering a more open and cooperative data-sharing environment, researchers can gain access to the necessary resources to develop and validate more robust multimodal integration models. This, in turn, can lead to improved diagnostic accuracy and patient outcomes, ultimately benefiting the healthcare sector.

In addition, the presence of outliers in the data can significantly impact model performance (Spasov et al., 2018). Thus, future research should prioritize data preprocessing techniques aimed at detecting and removing outliers from the dataset before model training. Outlier detection techniques such as *z*-score, isolation forest, or k-nearest neighbors can be employed to identify and remove outliers from the dataset before training the model. After removing outliers from the data, another challenge arises which is integration difficulties between high-dimensional medical images and low-dimensional EHR features (Xu et al., 2021). To tackle this challenge, future work should explore dimensionality reduction techniques such as PCA and autoencoders. These techniques can be employed to achieve dimensionality reduction while preserving the discriminative power of the data.

Therefore, future research efforts should focus on developing and improving data fusion methodologies that address challenges related to noisy or limited data, outlier detection, and integration difficulties. By overcoming these challenges, future frameworks for multimodal deep learning in clinical decision support can significantly enhance diagnostic accuracy, treatment efficacy, and patient outcomes in healthcare settings.

## Conclusion

This paper presents a comprehensive analysis of methods for integrating multiple types of data in artificial intelligence models for healthcare. Our review includes 69 relevant publications from 2018 to 2024, offering an in-depth investigation of fusion techniques, such as incorporating medical images with organized and unorganized data, merging distinct image modalities, and amalgamating diverse characteristics. We highlight the utilization of data fusion approaches for different diseases, demonstrating how customized fusion strategies can effectively address specific diagnostic and therapeutic challenges. Focusing on these diseases provides a clearer understanding of the practical benefits of combining multiple data types in therapeutic settings. Our extensive review of contemporary data fusion technologies and their applications is a valuable resource for scholars and practitioners. By outlining the advantages and constraints of each method, we provide direction for future research aimed at creating and enhancing multimodal AI models in healthcare. Data fusion technologies are continually advancing and hold great potential for the future of healthcare. Advancements in this domain could improve the resilience, effectiveness, and precision of AI systems, enhancing patient outcomes and propelling medical science forward. The integration of diverse healthcare data from multiple sources is crucial for the advancement of AI model development. This paper enhances current knowledge by combining previous literature and examining different fusion strategies, providing a comprehensive understanding of the subject and establishing a foundation for future research focused on utilizing multimodal data to develop better healthcare solutions.

## Additional Information and Declarations

### Data Availability

The following information was supplied regarding data availability: This is a literature review.

## References

[ref-1] Abdelaziz M, Wang T, Elazab A (2021). Alzheimer’s disease diagnosis framework from incomplete multimodal data using convolutional neural networks. Journal of Biomedical Informatics.

[ref-2] Acosta JN, Falcone GJ, Rajpurkar P, Topol EJ (2022). Multimodal biomedical AI. Nature Medicine.

[ref-3] Akazawa M, Hashimoto K (2023). A multimodal deep learning model for predicting severe hemorrhage in placenta previa. Scientific Reports.

[ref-4] Behrad F, Abadeh MS (2022). An overview of deep learning methods for multimodal medical data mining. Expert Systems with Applications.

[ref-5] Bhagwat N, Viviano JD, Voineskos AN, Chakravarty MM, Alzheimer’s Disease Neuroimaging Initiative (2018). Modeling and prediction of clinical symptom trajectories in Alzheimer’s disease using longitudinal data. PLOS Computational Biology.

[ref-6] Cai G, Zhu Y, Wu Y, Jiang X, Ye J, Yang D (2023). A multimodal transformer to fuse images and metadata for skin disease classification. Visual Computer.

[ref-7] Caruso C, Guarrasi V, Cordelli E, Sicilia R, Gentile S, Messina L, Fiore M, Piccolo C, Zobel B, Iannello G, Ramella S, Soda P (2022). A multimodal ensemble driven by multiobjective optimisation to predict overall survival in non-small-cell lung cancer. Medical Image Computing and Computer-Assisted Intervention.

[ref-8] Chaganti S, Bermudez C, Mawn L, Lasko T, Landman B (2019). Contextual deep regression network for volume estimation in orbital CT.

[ref-9] Chen H, Guo H, Xing L, Chen D, Yuan T, Zhang Y, Zhang X (2023). Multimodal predictive classification of Alzheimer’s disease based on attention-combined fusion network: integrated neuroimaging modalities and medical examination data. IET Image Processing.

[ref-10] Chen R, Lu M, Weng W, Chen T, Williamson D, Manz T, Shady M, Mahmood F (2021). Multimodal co-attention transformer for survival prediction in gigapixel whole slide images.

[ref-11] Dai Y, Bai WH, Tang Z, Xu ZA, Chen WB (2021). Computer-aided diagnosis of alzheimer’s disease *via* deep learning models and radiomics method. Applied Sciences.

[ref-12] Dimitriadis SI, Liparas D, Tsolaki MN, Alzheimer’s Disease Neuroimaging Initiative (2018). Random forest feature selection, fusion and ensemble strategy: Combining multiple morphological MRI measures to discriminate among healhy elderly, MCI, cMCI and alzheimer’s disease patients: from the alzheimer’s disease neuroimaging initiative (ADNI) database. Journal of Neuroscience Methods.

[ref-13] Dipaola F, Gatti M, Levra A, Mene R, Shiffer D, Faccincani R, Raouf Z, Secchi A, Querini P, Voza A, Badalamenti S, Solbiati M, Costantino G, Savevski V, Furlan R (2023). Multimodal deep learning for COVID-19 prognosis prediction in the emergency department: a bi-centric study. Scientific Reports.

[ref-14] El-Ateif S, Idri A (2022). Single-modality and joint fusion deep learning for diabetic retinopathy diagnosis. Scientific African.

[ref-15] El-Sappagh S, Abuhmed T, Islam SMR, Kwak KS (2020). Multimodal multitask deep learning model for Alzheimer’s disease progression detection based on time series data. Neurocomputing.

[ref-16] Feng CY, Elazab A, Yang P, Wang TF, Zhou F, Hu HY, Xiao XH, Lei BY (2019). Deep learning framework for alzheimer’s disease diagnosis *via* 3D-CNN and FSBi-LSTM. IEEE Access.

[ref-17] Fu Y, Xue P, Li N, Zhao P, Xu Z, Ji H, Zhang Z, Cui W, Dong E (2021). Fusion of 3D lung CT and serum biomarkers for diagnosis of multiple pathological types on pulmonary nodules. Computer Methods and Programs in Biomedicine.

[ref-18] Gaw N, Yousefi S, Gahrooei MR (2022). Multimodal data fusion for systems improvement: a review. IISE Transactions.

[ref-19] Golovanevsky M, Eickhoff C, Singh R (2022). Multimodal attention-based deep learning for Alzheimer’s disease diagnosis. Journal of the American Medical Informatics Association.

[ref-20] Holste G, Dvd W, Pinckaers H, Yamashita R, Mitani A, Esteva A (2023). Improved multimodal fusion for small datasets with auxiliary supervision.

[ref-21] Hsieh C, Nobre I, Sousa S, Ouyang C, Brereton M, Nascimento J, Jorge J, Moreira C (2023). MDF-Net for abnormality detection by fusing X-rays with clinical data. Scientific Reports.

[ref-22] Hu P, Li X, Lu N, Dong K, Bai X, Liang T, Li J (2023). Prediction of new-onset diabetes after pancreatectomy with subspace clustering based multi-view feature selection. IEEE Journal of Biomedical and Health Informatics.

[ref-23] Huang C, Chen W, Liu B, Yu R, Chen X, Tang F, Liu J, Lu W (2022). Transformer-based deep-learning algorithm for discriminating demyelinating diseases of the central nervous system with neuroimaging. Frontiers in Immunology.

[ref-24] Huang SC, Pareek A, Seyyedi S, Banerjee I, Lungren MP (2020a). Fusion of medical imaging and electronic health records using deep learning: a systematic review and implementation guidelines. NPJ Digital Medicine.

[ref-25] Huang SC, Pareek A, Zamanian R, Banerjee I, Lungren MP (2020b). Multimodal fusion with deep neural networks for leveraging CT imaging and electronic health record: a case-study in pulmonary embolism detection. Scientific Reports.

[ref-26] Hsu MY, Chiou JY, Liu JT, Lee CM, Lee YW, Chou CC, Lo SC, Kornelius E, Yang YS, Chang SY, Liu YC, Huang CN, Tseng VS (2021). Deep learning for automated diabetic retinopathy screening fused with heterogeneous data from EHRs can lead to earlier referral decisions. Translational Vision Science & Technology.

[ref-27] Jacenków G, O’Neil AQ, Tsaftaris SA (2022). Indication as prior knowledge for multimodal disease classification in chest radiographs with transformers.

[ref-28] Joo S, Ko ES, Kwon S, Jeon E, Jung H, Kim JY, Chung MJ, Im YH (2021). Multimodal deep learning models for the prediction of pathologic response to neoadjuvant chemotherapy in breast cancer. Scientific Reports.

[ref-29] Jin K, Yan Y, Chen M, Wang J, Pan X, Liu X, Liu M, Lou L, Wang Y, Ye J (2022). Multimodal deep learning with feature level fusion for identification of choroidal neovascularization activity in age-related macular degeneration. Acta Ophthalmologica.

[ref-30] Jujjavarapu C, Suri P, Pejaver V, Friedly J, Gold L, Meier E, Cohen T, Mooney S, Heagerty P, Jarvik J (2023). Predicting decompression surgery by applying multimodal deep learning to patients’ structured and unstructured health data. BMC Medical Informatics and Decision Making.

[ref-31] Kadri R, Bouaziz B, Tmar M, Gargouri F (2023). Efficient multimodel method based on transformers and CoAtNet for Alzheimer’s diagnosis. Digital Signal Processing.

[ref-32] Kharazmi P, Kalia S, Lui H, Wang Z, Lee T (2018). A feature fusion system for basal cell carcinoma detection through data-driven feature learning and patient profile. Skin Research and Technology.

[ref-33] Kumar S, Chaube MK, Alsamhi SH, Gupta SK, Guizani M, Gravina R, Fortino G (2022a). A novel multimodal fusion framework for early diagnosis and accurate classification of COVID-19 patients using X-ray images and speech signal processing techniques. Computer Methods and Programs in Biomedicine.

[ref-34] Kumar S, Gupta SK, Kumar V, Kumar M, Chaube MK, Naik NS (2022b). Ensemble multimodal deep learning for early diagnosis and accurate classification of COVID-19. Computers & Electrical Engineering.

[ref-35] Lee Y, Cha J, Shim I, Park W, Kang S, Lim D, Won H (2023). Multimodal deep learning of fundus abnormalities and traditional risk factors for cardiovascular risk prediction. Npj Digital Medicine.

[ref-36] Li K, Chen C, Cao W, Wang H, Han S, Wang R, Ye Z, Wu Z, Wang W, Cai L, Ding D, Yuan Z (2023a). DeAF: a multimodal deep learning framework for disease prediction. Computers in Biology and Medicine.

[ref-37] Li Z, Chen W, Ju Y, Chen Y, Hou Z, Li X, Jiang Y (2023b). Bone age assessment based on deep neural networks with annotation-free cascaded critical bone region extraction. Frontiers in Artificial Intelligence.

[ref-38] Li HM, Fan Y (2019). EARLY prediction of alzheimer’s disease dementia based on baseline hippocampal mri and 1-year follow-up cognitive measures using deep recurrent neural networks.

[ref-39] Lin WM, Gao QQ, Yuan JN, Chen ZY, Feng CW, Chen WS, Du M, Tong T (2020). Predicting alzheimer’s disease conversion from mild cognitive impairment using an extreme learning machine-based grading method with multimodal data. Frontiers in Aging Neuroscience.

[ref-40] Lin MQ, Wang S, Ding Y, Zhao LH, Wang F, Peng YF, Soc IC (2021). An empirical study of using radiology reports and images to improve ICU-mortality prediction.

[ref-41] Lipkova J, Chen RJ, Chen BW, Lu MY, Barbieri M, Shao D, Vaidya AJ, Chen CK, Zhuang LT, Williamson DFK, Shaban M, Chen TY, Mahmood F (2022). Artificial intelligence for multimodal data integration in oncology. Cancer Cell.

[ref-42] Liu JJ, Chen YC, Lan L, Lin BL, Chen WJ, Wang MH, Li R, Yang YJ, Zhao B, Hu ZL, Duan YX (2018). Prediction of rupture risk in anterior communicating artery aneurysms with a feed-forward artificial neural network. European Radiology.

[ref-43] Lu PX, Hu LT, Mitelpunkt A, Bhatnagar S, Lu L, Liang HY (2024). A hierarchical attention-based multimodal fusion framework for predicting the progression of Alzheimer’s disease. Biomedical Signal Processing and Control.

[ref-44] Mammoottil MJ, Kulangara LJ, Cherian AS, Mohandas P, Hasikin K, Mahmud M (2022). Detection of breast cancer from five-view thermal images using convolutional neural networks. Journal of Healthcare Engineering.

[ref-45] Mohsen F, Ali H, Hajj NEl, Shah Z (2022). Artificial intelligence-based methods for fusion of electronic health records and imaging data. Scientific Reports.

[ref-46] Mokni R, Gargouri N, Damak A, Sellami D, Feki W, Mnif Z (2021). An automatic computer-aided diagnosis system based on the multimodal fusion of breast cancer (MF-CAD). Biomedical Signal Processing and Control.

[ref-47] Moshawrab M, Adda M, Bouzouane A, Ibrahim H, Raad A (2023). Reviewing multimodal machine learning and its use in cardiovascular diseases detection. Electronics.

[ref-48] M’Sabah CEL, Bouziane A, Ferdi Y (2021). A survey on deep learning methods for cancer diagnosis using multimodal data fusion.

[ref-49] Nie D, Lu JF, Zhang H, Adeli E, Wang J, Yu ZD, Liu LY, Wang Q, Wu JS, Shen DG (2019). Multi-channel 3D deep feature learning for survival time prediction of brain tumor patients using multi-modal neuroimages. Scientific Reports.

[ref-50] Odusami M, Maskeliunas R, Damasevicius R, Misra S (2023). Explainable deep-learning-based diagnosis of alzheimer’s disease using multimodal input fusion of PET and MRI images. Journal of Medical and Biological Engineering.

[ref-51] Oh S, Kang S, Oh I, Kim M (2023). Deep learning model integrating positron emission tomography and clinical data for prognosis prediction in non-small cell lung cancer patients. BMC Bioinformatics.

[ref-52] Purwar S, Tripathi RK, Ranjan R, Saxena R (2020). Detection of microcytic hypochromia using cbc and blood film features extracted from convolution neural network by different classifiers. Multimedia Tools and Applications.

[ref-53] Puyol-Anton E, Sidhu BS, Gould J, Porter B, Elliott MK, Mehta V, Rinaldi CA, King AP (2022). A multimodal deep learning model for cardiac resynchronisation therapy response prediction. Medical Image Analysis.

[ref-54] Qiu W, Yang J, Wang B, Yang M, Tian G, Wang P, Yang J (2022). Evaluating the microsatellite instability of colorectal cancer based on multimodal deep learning integrating histopathological and molecular data. Frontiers in Oncology.

[ref-55] Rahim N, Abuhmed T, Mirjalili S, El-Sappagh S, Muhammad K (2023a). Time-series visual explainability for Alzheimer’s disease progression detection for smart healthcare. Alexandria Engineering Journal.

[ref-56] Rahim N, El-Sappagh S, Ali S, Muhammad K, Del Ser J, Abuhmed T (2023b). Prediction of Alzheimer’s progression based on multimodal Deep-Learning-based fusion and visual Explainability of time-series data. Information Fusion.

[ref-57] Reda I, Khalil A, Elmogy M, El-Fetouh AAbou, Shalaby A, Abou El-Ghar M, Elmaghraby A, Ghazal M, El-Baz A (2018). Deep learning role in early diagnosis of prostate cancer. Technology in Cancer Research & Treatment.

[ref-58] Schilcher J, Nilsson A, Andlid O, Eklund A (2024). Fusion of electronic health records and radiographic images for a multimodal deep learning prediction model of atypical femur fractures. Computers in Biology and Medicine.

[ref-59] Schulz S, Woerl A, Jungmann F, Glasner C, Stenzel P, Strobl S, Fernandez A, Wagner D, Haferkamp A, Mildenberger P, Roth W, Foersch S (2021). Multimodal deep learning for prognosis prediction in renal cancer. Frontiers in Oncology.

[ref-60] Shetty S, Ananthanarayana VS, Mahale A (2023). Multimodal medical tensor fusion network-based DL framework for abnormality prediction from the radiology CXRs and clinical text reports. Multimedia Tools and Applications.

[ref-61] Silva L, Rohr K (2020). Pan-cancer prognosis prediction using multimodal deep learning.

[ref-62] Spasov SE, Passamonti L, Duggento A, Lio P, Toschi N (2018). A multi-modal convolutional neural network framework for the prediction of alzheimer’s disease.

[ref-63] Stahlschmidt SR, Ulfenborg B, Synnergren J (2022). Multimodal deep learning for biomedical data fusion: a review. Briefings in Bioinformatics.

[ref-64] Sun ZY, Lin MQ, Zhu QQ, Xie QQ, Wang F, Lu ZY, Peng YF (2023). A scoping review on multimodal deep learning in biomedical images and texts. Journal of Biomedical Informatics.

[ref-65] Tan PX, Huang W, Wang LL, Deng GH, Yuan Y, Qiu SL, Ni D, Du SS, Cheng J (2022). Deep learning predicts immune checkpoint inhibitor-related pneumonitis from pretreatment computed tomography images. Frontiers in Physiology.

[ref-66] Tang C, Wei M, Sun J, Wang S, Zhang Y, Alzheimers Dis Neuroimaging I (2023). CsAGP: detecting Alzheimer’s disease from multimodal images via dual-transformer with cross-attention and graph pooling. Journal of King Saud University-Computer and Information Sciences.

[ref-67] Wang Z, Li R, Wang M, Li A (2021). GPDBN: deep bilinear network integrating both genomic data and pathological images for breast cancer prognosis prediction. Bioinformatics.

[ref-68] Wei W, Jia G, Wu Z, Wang T, Wang H, Wei K, Cheng C, Liu Z, Zuo C (2023). A multidomain fusion model of radiomics and deep learning to discriminate between PDAC and AIP based on <SUP>18</SUP>F-FDG PET/CT images. Japanese Journal of Radiology.

[ref-69] Xu M, Ouyang L, Han L, Sun K, Yu TT, Li Q, Tian H, Safarnejad L, Zhang HD, Gao Y, Bao FS, Chen YF, Robinson P, Ge YR, Zhu BL, Liu J, Chen S (2021). Accurately differentiating between patients with COVID-19, patients with other viral infections, and healthy individuals: multimodal late fusion learning approach. Journal of Medical Internet Research.

[ref-70] Yala A, Lehman C, Schuster T, Portnoi T, Barzilay R (2019). A deep learning mammography-based model for improved breast cancer risk prediction. Radiology.

[ref-71] Yan R, Zhang F, Rao X, Lv Z, Li J, Zhang L, Liang S, Li Y, Ren F, Zheng C, Liang J (2021). Richer fusion network for breast cancer classification based on multimodal data. BMC Medical Informatics and Decision Making.

[ref-72] Yang J, Ju J, Guo L, Ji B, Shi S, Yang Z, Gao S, Yuan X, Tian G, Liang Y, Yuan P (2022). Prediction of HER2-positive breast cancer recurrence and metastasis risk from histopathological images and clinical information via multimodal deep learning. Computational and Structural Biotechnology Journal.

[ref-73] Yao J, Lei Z, Yue W, Feng B, Li W, Ou D, Feng N, Lu Y, Xu J, Chen W, Yang C, Wang L, Wang L, Liu J, Wei P, Xu H, Xu D (2022). DeepThy-Net: a multimodal deep learning method for predicting cervical lymph node metastasis in papillary thyroid cancer. Advanced Intelligent Systems.

[ref-74] Yao D, Xu Z, Lin Y, Zhan Y (2023). Accurate and intelligent diagnosis of pediatric pneumonia using X-ray images and blood testing data. Frontiers in Bioengineering and Biotechnology.

[ref-75] Yap J, Yolland W, Tschandl P (2018). Multimodal skin lesion classification using deep learning. Experimental Dermatology.

[ref-76] Yoo Y, Tang LYW, Li DKB, Metz L, Kolind S, Traboulsee AL, Tam RC (2019). Deep learning of brain lesion patterns and user-defined clinical and MRI features for predicting conversion to multiple sclerosis from clinically isolated syndrome. Computer Methods in Biomechanics and Biomedical Engineering-Imaging and Visualization.

[ref-77] Yuan C, Shi Q, Huang X, Wang L, He Y, Li B, Zhao W, Qian D (2023). Multimodal deep learning model on interim <SUP>18</SUP>F FDG PET/CT for predicting primary treatment failure in diffuse large B-cell lymphoma. European Radiology.

[ref-78] Zhang G, He X, Li D, Tian C, Wei B (2022). Automated screening of COVID-19-based tongue image on chinese medicine. Biomed Research International.

[ref-79] Zhang K, Lincoln JA, Jiang XQ, Bernstam EV, Shams S (2023). Predicting multiple sclerosis severity with multimodal deep neural networks. BMC Medical Informatics and Decision Making.

[ref-80] Zhao D, Homayounfar M, Zhen Z, Wu M, Yu S, Yiu K, Vardhanabhuti V, Pelekos G, Jin L, Koohi-Moghadam M (2022). A multimodal deep learning approach to predicting systemic diseases from oral conditions. Diagnostics.

[ref-81] Zheng WB, Yan L, Gou C, Zhang ZC, Zhang JJ, Hu M, Wang FY (2021). Pay attention to doctor-patient dialogues: multi-modal knowledge graph attention image-text embedding for COVID-19 diagnosis. Information Fusion.

[ref-82] Zhou JZ, Zhang XM, Zhu ZW, Lan XY, Fu LK, Wang HX, Wen HC (2022). Cohesive multi-modality feature learning and fusion for COVID-19 patient severity prediction. IEEE Transactions on Circuits and Systems for Video Technology.

